# Delineating the Extracellular Water-Accessible Surface of the Proton-Coupled Folate Transporter

**DOI:** 10.1371/journal.pone.0078301

**Published:** 2013-10-18

**Authors:** Phaneendra Kumar Duddempudi, Raman Goyal, Swapneeta Sanjay Date, Michaela Jansen

**Affiliations:** 1 Department of Pharmacology and Neuroscience, School of Medicine, Texas Tech University Health Sciences Center, Lubbock, Texas, United States of America; 2 Center for Membrane Protein Research, School of Medicine, Texas Tech University Health Sciences Center, Lubbock, Texas, United States of America; 3 Department of Cell Physiology and Molecular Biophysics, School of Medicine, Texas Tech University Health Sciences Center, Lubbock, Texas, United States of America; University of Cambridge, United Kingdom

## Abstract

The proton-coupled folate transporter (PCFT) was recently identified as the major uptake route for dietary folates in humans. The three-dimensional structure of PCFT and its detailed interplay with function remain to be determined. We screened the water-accessible extracellular surface of *Hs*PCFT using the substituted-cysteine accessibility method, to investigate the boundaries between the water-accessible surface and inaccessible buried protein segments. Single-cysteines, engineered individually at 40 positions in a functional cysteine-less *Hs*PCFT background construct, were probed for plasma-membrane expression in *Xenopus* oocytes with a bilayer-impermeant primary-amine-reactive biotinylating agent (sulfosuccinimidyl 6-(biotinamido) hexanoate), and additionally for water-accessibility of the respective engineered cysteine with the sulfhydryl-selective biotinylating agent 2-((biotinoyl)amino)ethyl methanethiosulfonate. The ratio between Cys-selective over amine-selective labeling was further used to evaluate three-dimensional models of *Hs*PCFT generated by homology / threading modeling. The closest homologues of *Hs*PCFT with a known experimentally-determined three-dimensional structure are all members of one of the largest membrane protein super-families, the major facilitator superfamily (MFS). The low sequence identity - 14% or less – between *Hs*PCFT and these templates necessitates experiment-based evaluation and model refinement of homology / threading models. With the present set of single-cysteine accessibilities, the models based on GlpT and PepT_St_ are most promising for further refinement.

## Introduction

Folate (vitamin B9) belongs to the water-soluble B vitamin group. The name is derived from the Latin word for leaf (*folium*), as green leafy vegetables are naturally rich in folate. Oxidized and polyglutamate forms of folate are dietary active, but must be deconjugated to remove glutamic acid moieties and reduced to dihydrofolate and tetrahydrofolate to confer biological activity. These reduced compounds play important roles in one-carbon metabolism pathways such as biosynthesis of nucleic-acids and also in synthesis and breakdown of amino acids. Common to all vitamins, folates cannot be synthesized by mammals, and are therefore considered vital nutrients. Folates are highly absorbed from dietary sources in the intestine by specialized membrane proteins. Folate deficiency in pregnant women increases the risk for the development of neural tube defects (NTDs) in the offspring [1]. Deficiency in adults may result in various pathological conditions including cardiovascular diseases and cancer [2,3]. However, adverse effects of high folate status include, decreased natural killer cell cytotoxicity, as well as progression and growth of preneoplastic cells [4]. Due to its hydrophilic anionic structure, folic acid cannot freely diffuse across lipid bilayers, and efficient uptake into cells must therefore be facilitated by specialized membrane proteins. Specific transmembrane proteins that mediate folate uptake into cells are the proton-coupled folate transporter (PCFT), the reduced-folate carrier (RFC), and folate receptors (FRα and FRβ)[5-7]. These membrane proteins transport folate by different mechanisms and with different affinities and substrate specificities. PCFT and RFC are secondary-active transporters with optimum transport activity at acidic and neutral pH, respectively, while FRs are glycosylphosphatidylinositol (GPI) linked proteins and transport folates via endocytosis [8,9]. 

PCFT is highly expressed in the brush-border membrane of the proximal part of the small intestine, where folates are maximally absorbed [6]. PCFT also transports folates from the blood into the cerebrospinal fluid through its expression on the basolateral membrane of ependymal cells in the choroid plexus [6,10]. Loss-of-function mutations of PCFT in humans disrupt these physiological roles and result in the development of a rare recessive autosomal disorder known as Hereditary Folate Malabsoption (HFM)[6]. Several mutations in the *Hs*PCFT gene that result in HFM have been identified and characterized. Implications of HFM are low levels of folate in the blood and CNS, which result in megaloblastic anemia, immune deficiency, and neurological manifestations, including seizures, developmental delays, cognitive and motor impairment. PCFT expression was identified in various solid-tumor cell lines, where its acidic pH optimum, together with the acidic microenvironment surrounding these cells, facilitates transport of folates into solid tumors [11]. Novel antifolates are synthesized that can be transported effectively through PCFT into human solid tumors [11].

PCFT belongs to the major facilitator superfamily (MFS) that comprises over 50 families. X-ray structures for prokaryotic members of six MFS families that utilize proton-cotransport and for one exchanger are currently available, but structural information about PCFT is very limited at present. PCFT is functional as a monomer in plasma membranes [12]. Intracellular higher order oligomeric states have been observed under conditions of transient overexpression [13]. Hydrophobicity analysis of *Hs*PCFT’s amino acid sequence indicated that PCFT is composed of 12 transmembrane a-helices (TMI to TMXII) which was confirmed using a single-Cys in each loop between transmembrane segments, using the substituted-cysteine accessibility method (SCAM) [14,15]. PCFT contains two consensuses N-glycosylation sites (N58 and N68), located in the first extracellular loop between transmembrane segments 1 and 2 [16]. Immunohistochemical analysis investigating the accessibility of an engineered HA epitope attached to PCFT showed that both N- and C-termini are located on the cytoplasmic side [17]. Site-directed mutagenesis of residues identified in loss-of-function mutations of the *Hs*PCFT gene involved in HFM have identified several functionally or/and structurally important elements in *Hs*PCFT [18-27]. Server-based methods for homology-prediction and homology model-generation based on transporters with experimentally-determined - for example X-ray – three-dimensional structure can be applied to PCFT. However, due to the low sequence identity (14% or less) with identified templates, any model will require careful experimental validation. Previously, three-dimensional PCFT models were constructed using X-ray-derived structures of transporters with predicted structural homology, the glycerol-3-phosphate transporter (GlpT, 1pw4) or a multidrug transporter (EmrD, 2gfp) as templates [24,28]. Importantly, at present, six out of nine server-identified templates have been structurally characterized after the previous homology models were generated.

Accordingly, we designed the present study to generate and validate PCFT homology models. We utilized cysteine-scanning in combination with the substituted-cysteine accessibility method (SCAM) to investigate the extent of accessibility of all twelve transmembrane segments towards their respective extracellular ends and of the predicted extracellular loops towards the extracellular side of the plasma membranes. Initially, we manually generated a secondary structure model based on a three-dimensional homology model of PCFT built on the X-ray structure of GlpT as a template. We engineered 40 PCFT constructs, each containing a single-Cys in or close to an extracellular loop based on its location in our secondary structure model of PCFT. The plasma-membrane expression of each single-Cys construct was probed with sulfosuccinimidyl 6-(biotinamido) hexanoate (Sulfo-NHS-LC-biotin), and the accessibility of each engineered Cys was tested utilizing the sulfhydryl-selective reagent 2-((biotinoyl)amino)ethyl methanethiosulfonate (MTSEA-biotin). The Cys-accessibility normalized against the primary amine-accessibility was then used to evaluate nine homology models generated by HHpred/MODELLER and LOMETS/MODELLER. We identified the models based on GlpT and PepT_St_ most suitable for further studies.

## Materials and Methods

### Reagents

Sulfosuccinimidyl 6-(biotinamido) hexanoate (sulfo-NHS-LC-biotin) and High Capacity NeutrAvidin Agarose Resin were purchased from Thermo Scientific (Rockford, IL, USA). MTSEA-biotin was obtained from Toronto Research Chemicals (Toronto, Ontario, Canada).

### Site-directed mutagenesis of PCFT

A ctV5-*Hs*PCFT-CYS- construct in the pXOON vector [29] was constructed by replacing all seven endogenous Cys residues in ctV5-*Hs*PCFT pXOON (PCFT wild-type with a C-terminal V5-epitope tag, GKPIPNPLLGLDST) with serine, using the QuikChange^TM^ Lightning Site-Directed Mutagenesis kit (Agilent, Santa Clara, CA, USA). This construct was used as a template to generate 40 single-Cys mutants by site-directed mutagenesis using the QuikChange^TM^ kit (Agilent). We tried to engineer the Cys at positions such that the mutations were conserved. The mutagenesis primers were generated based on the manufacturer’s recommendations and sequences are available upon request. A total of 40 residues were mutated to Cys, one at a time, in all six predicted extracellular loops, and in the predicted extracellular ends of the transmembrane segments. All mutations were confirmed by DNA sequencing of the entire gene (Genewiz, South Plainfield, NJ, USA).

### *Xenopus laevis* oocytes

Large, adult lab-bred female *Xenopus laevis* were purchased from *Xenopus* Express (Hamosassa, FL, USA). Oocytes were harvested from tricaine-anesthetized frogs, and washed in oocyte Ringer’s buffer, OR2 (in mM: 82.5 NaCl, 2 KCl, 1 MgCl_2_, and 5 HEPES; pH adjusted to 7.5 with NaOH) before treatment with collagenase (2 mg/ml type 1A, Sigma, St. Louis, MO, USA) in the same buffer for 45-75 minutes at room temperature. Oocytes were treated thoroughly with OR2 containing Ca for three 45-minute intervals with media changes in between each incubation. Oocytes were then maintained in standard oocyte saline (SOS) medium (in mM: 100 NaCl, 2 KCl, 1.8 CaCl_2_, 1 MgCl_2_, and 5 mM HEPES, pH 7.5), supplemented with 1% antibiotic–antimycotic (100x) liquid (10,000 IU/ml penicillin, 10,000 µg/ml streptomycin, and 25 µg/ml amphotericin B; Invitrogen, Carlsbad, CA, USA) and 5% horse serum (Sigma). Oocytes were injected with 50 ng of in-vitro synthesized mRNA 12 to 24 h after harvest, and subsequently incubated in horse serum media for 4-10 days at 16-18°C.

### Radiosubstrate Uptake by PCFT-expressing *X. laevis* oocytes

PCFT mediated uptake of [^3^H]folic acid (Moravek Biochemicals, Inc., Brea, CA) into *X. laevis* oocytes was determined in MES buffered saline (MBS) buffer (140 mM NaCl, 2.8 mM KCl, 2 mM MgCl_2_, 1 mM CaCl_2_, 10 mM MES, pH 5.5). Transport of folic acid through PCFT is proton-coupled and therefore facilitated by acidic pH. Therefore, uptake was studied at pH 5.5 [6]. Oocytes were washed 3-4 times with Hepes buffered saline (HBS) buffer (140 mM NaCl, 2.8 mM KCl, 2 mM MgCl_2_, 1 mM CaCl_2_, 10 mM HEPES, pH 7.4). Uptake was initiated by placing 3-5 oocytes into MBS buffer (pH 5.5) containing a 0.015 μM concentration of [^3^H]folic acid. After incubation for 10 min at room temperature, uptake was halted by 5-6 rapid washes with cold MBS buffer (pH 5.5). Oocytes were individually solubilized in 300 μl of 5% SDS for 60 minutes to overnight, and uptake of radiolabeled substrate was determined with a Packard 1900 TR liquid scintillation analyzer or a Beckman LS 6500 Scintillation Counter. To evaluate non-PCFT mediated folic acid uptake in oocytes, control experiments were performed with uninjected oocytes. Comparison of uptake in noninjected vs. water-injected oocytes showed no significant differences (data not shown). Uptake expressed in picomoles of [^3^H]folic acid per oocyte. 

### Biotinylation of *Xenopus laevis* oocytes with Sulfo-NHS-LC-biotin

4-5 days after injection, oocytes were washed three times with 6 ml of calcium-free OR-2. Surface proteins were biotinylated with 0.5 mg/ml sulfo-NHS-LC-biotin for 30 minutes at room temperature. Then the oocytes were washed three times with 6 ml of calcium-free OR-2 solution. The excess amount of sulfo-NHS-LC-biotin was quenched by incubating the oocytes for 10 minutes in buffer H (100 mM NaCl, 20 mM Tris, pH 7.4). The oocytes were triturated at 4°C in 20 μl/oocyte buffer H++ (buffer H with 1% Triton X-100, 0.5% deoxycholate, and 1x HALT protease inhibitor cocktail, Thermo Scientific), solubilized by rotating at 4°C for 60 minutes and spun at 21,000 g for 10 minutes at 4°C. After carefully removing the debris and yolk, the supernatant was again spun at 21,000 g for 10 minutes at 4°C to remove any residual debris and yolk. To isolate biotinylated proteins, the supernatant was incubated with prewashed and buffer H++-equilibrated neutravidin beads for 2 hours at 4°C. After incubation the beads were spun at 2,500 g for 2.5 minutes at room temperature to remove unbound proteins. The beads were washed three times with 1ml of buffer H++, with the last wash supplemented with 2% SDS. The biotinlyated proteins were eluted from the beads by adding 60 µl of 4X SDS-sample buffer with DTT. Samples were loaded on 4-15 % Precast criterion gels (Bio-rad), transferred to PVDF membranes, and probed with V5 HRP antibody (1:5,000 in 5% milk for 4 hours at room temperature). 

### Biotinylation of *Xenopus laevis* oocytes with MTSEA-biotin

The procedure followed for Cys-biotinylation was comparable to the one described for primary amine-biotinylation described above, except that MTSEA-biotin was used. Excess MTSEA-biotin was removed by washing extensively with OR-2. 

### Western blot analysis

Densitometry analysis of the Western blots was performed with the AlphaEase^FC^ software after image capture with the Fluor Chem^SP^ Imager (Alpha Innotech Co., San Leandro, CA). For each mutant we determined the ratios between Western blot signals with ImageJ after labeling with the Cys-selective label (MTSEA-biotin) over the Lys-selective label (sulfo-NHS-LC-biotin) to quantify Cys-accessibility over surface expression. The accessibility ratios were binned into three groups with regard to the accessibility of the respective Cys: Cys not accessible (ratio ≤ 0.3), Cys medium accessible (0.3 < ratio < 1.0), Cys highly accessible (ratio ≥ 1.0). 

### Statistical Analyses

Data shown are means ± SEM. One-way ANOVA with Bonferroni’s multiple comparison test or unpaired *t*-test (GraphPad Prism v5.0 software) was used to calculate statistical difference in [^3^H]folic acid uptake between *Hs*PCFT wild-type and *Hs*PCFT-CYS- injected oocytes. 

### Homology Modeling of PCFT

The HHpred server together with MODELLER (http://toolkit.tuebingen.mpg.de/hhpred) was used to search for remote homologues of PCFT (query date: Apr 26, 2013, GenBank accession number NP_NM_080669.4) with experimentally-determined crystal structures in the PDB database and to generate structural models using default parameters [30]. Similarly, LOMETS/MODELLER (http://zhanglab.ccmb.med.umich.edu/LOMETS/) was used to generate additional models.

## Results

### Homology Modeling of PCFT

**Figure 1:**
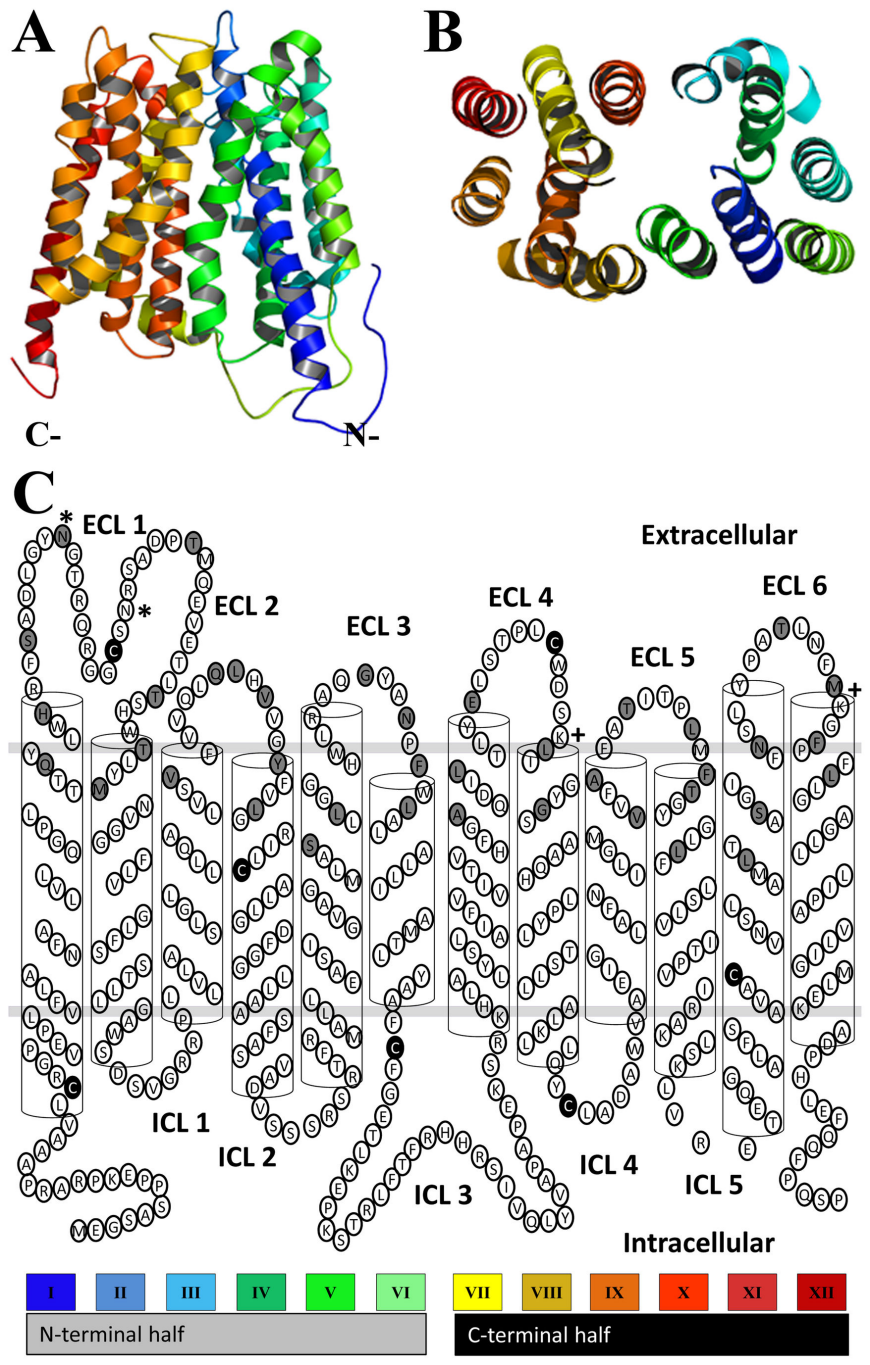
Initial modeling of PCFT. (A) A three-dimensional homology model of PCFT was built by the SWISS-MODEL server using an HHpred generated alignment with GlpT as input and the crystal structure of GlpT as a template. View parallel to the membrane showing all twelve transmembrane segments, color-coding as in panel C. (B) View perpendicular to the membrane looking into the predicted aqueous translocation pathway from the extracellular side. (C) Cartoon representation of the secondary structure of PCFT generated from the homology model by extrapolating information regarding the extent and orientation of transmembrane segments and loops. Gray-filled circles represent residues individually mutated to Cys. Black-filled circles identify endogenous Cys that were mutated to Ser. Numbering of extracellular loops (ECL) and intracellular loops (ICL) as indicated. Glycosylation sites indicated by “*”, and ECL surface Lys by “+”. Note, that the two Cys in ECL1 and ECL4 were also used as single-Cys mutants.

HHpred identified nine hits with ≥ 99.9 % probability [31,32]. Six hits are *E. coli* proteins, glycerol-3-phosphate transporter (GlpT, PDB # 1pw4)[33], multidrug resistance protein (EmrD, PDB # 2gfp)[34], L-fucose-proton symporter (FucP, PDB # 3o7q)[35], D-Xylose-proton symporter (XylE, PDB # 4gc0)[36], lactose permease (LacY, PDB # 2cfq)[37], nitrite extrusion protein (NarU, PDB # 4iu9)[38]. Two more hits are of prokaryotic origin, tripeptide-proton symporter (PepT_St_, PDB # 4aps)[39], and proton-peptide symporter (PepT_So_, PDB # 2xut)[40], and one hit of eukaryotic origin, phosphate transporter (PipT, PDB # 4j05)[41]. The E-values ranged between 7.5x10^-24^ to 1.8x10^-38^, and sequence identities between 9 and 14 % (Table 1). LOMETS identified the following templates with high confidence scores: GlpT, LacY, and EmrD. GlpT scored as the best template in HHpred and several sub-algorithms of LOMETS and therefore we chose it as the most suitable template to construct an initial homology model of PCFT. The alignment between PCFT and GlpT obtained from the HHpred server was used as input for structure modeling with the SWISS-MODEL server to generate a three-dimensional homology model of PCFT [42,43] that was subsequently used to generate a two-dimensional secondary structure of PCFT (Figure 1). For all identified templates default server settings were used to generate homology models with MODELLER.

**Table 1:** HHpred and LOMETS identified homologues of PCFT.

**Gene**	**MFS member**	**E-value**	**% identity**	**PDB #^@^**	**Resolution [Å]**	**Release date MM/YYYY**
GlpT	glycerol-3-phosphate transporter	3.7 x 10^-37^	14	1pw4^%,&^	3.30	08/2003
EmrD	multidrug resistance protein	1.8 x 10^-38^	13	2gfp^%,&^	3.50	05/2006
PipT	phosphate transporter	4.4 x 10^-32^	11	4j05^%^	2.90	04/2013
FucP	fucose-proton symporter	3.9 x 10^-29^	13	3o7q^%^	3.20	09/2010
XylE	D-Xylose-proton symporter	3.5 x 10^-28^	12	4gc0^%^	2.60	10/2012
LacY	lactose permease	7.4 x 10^-31^	12	2cfq^%^	2.95	03/2006
				1pv6^&^	3.50	08/2003
NarU	nitrite extrusion protein	3.1 x 10^-29^	9	4iu9^%^	3.01	04/2013
PepT_St_	tripeptide-proton symporter	4.4 x 10^-25^	14	4aps^%,&^	3.30	06/2012
PepT_So_	proton-peptide symporter	7.5 x 10^-24^	13	2xut^%^	3.62	12/2010

@ PDB # for homologues identified by HHpred are marked with “%” and by LOMETS by “&”.

### Cys-less PCFT is expressed in plasma membranes and functional

**Figure 2:**
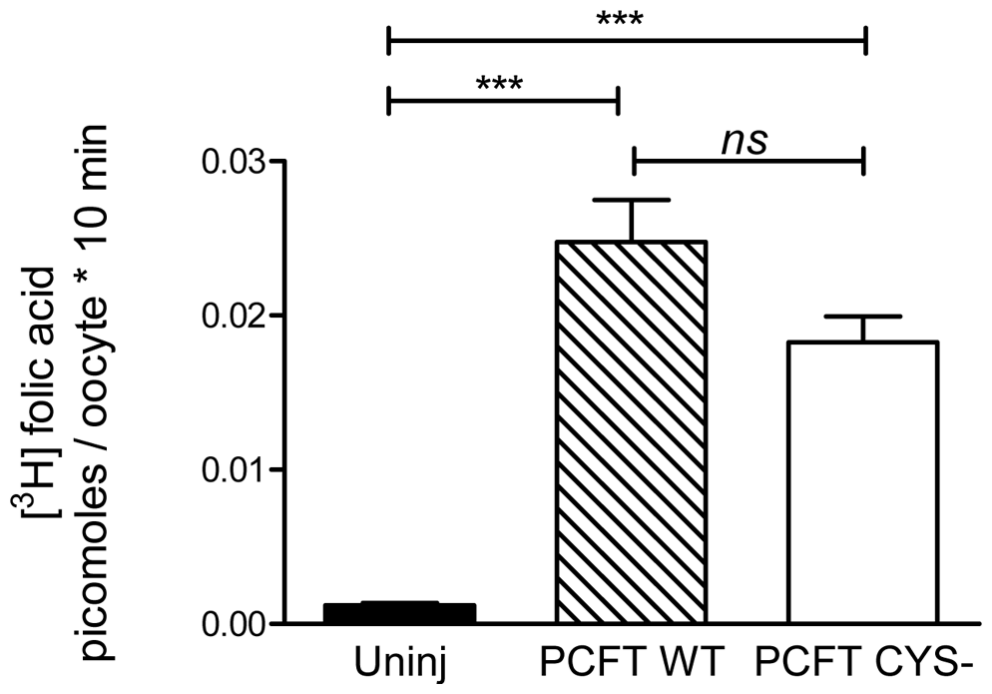
Transport activity of wild-type (WT) and Cys-less (CYS-) ct-V5-HsPCFT. Five days after the oocytes were injected with WT PCFT and PCFT CYS- mRNA, the uptake of 0.015 µM [^3^H]folic acid was measured at pH 5.5 over 10 minutes. Transport of folic acid through PCFT is proton-coupled and therefore facilitated by acidic pH. Therefore, uptake was studied at pH 5.5 [6]. Uninjected or water-injected oocytes (Uninj) were used as negative controls. Data represent the mean ± SEM from three different batches of oocytes. One-way ANOVA with Bonferroni’s multiple comparison test results shown. *** indicates p < 0.001; ns indicates not significant. Wild-type and Cys- are also not significantly different when analyzed with unpaired t test.

A Cys-less functional human PCFT (*Hs*PCFT) construct with a C-terminal hemagglutinin-tag (HA) and with all seven endogenous Cys replaced by Ser has been previously expressed in HeLa cells using the vector pcDNA3.1 [15,16]. We have generated a *Hs*PCFT construct with a C-terminal V5 epitope tag using the vector pXOON (ct-V5-*Hs*PCFT)[12]. We substituted all seven endogenous Cys in our ct-V5-*Hs*PCFT wild-type construct in the pXOON vector with Ser to obtain a Cys-less PCFT construct (ct-V5-*Hs*PCFT-Cys-, short PCFT-Cys-) (Figure 1). We expressed ct-V5-*Hs*PCFT with and without all endogenous Cys in Stage V *X. laevis* oocytes by micro-injecting the corresponding RNA. We first investigated if the Cys-less construct ct-V5-*Hs*PCFT-Cys- was expressed in plasma membranes of *X. laevis* oocytes by surface-biotinylating plasma-membrane proteins with membrane-impermeant sulfo-NHS-LC biotin. The biotinylated proteins were isolated with avidin beads, separated by SDS PAGE, transferred to PVDF membranes and probed with an antibody against the C-terminal V5 epitope tag. We found ct-V5-*Hs*PCFT and ct-V5-*Hs*PCFT-Cys- to be robustly expressed at the plasma membrane. To ascertain that the construct retained its ability to translocate folic acid across the plasma membrane facilitated by a pH gradient, we performed uptake studies with [^3^H]folic acid at pH 5.5 (Figure 2). No statistical significant difference between [^3^H]folic acid influx mediated by *Hs*PCFT wild-type and *Hs*PCFT-Cys- injected oocytes was observed. 

### Extracellular accessibility of single-Cys PCFT constructs with sulfo-NHS-LC-biotin and MTSEA-biotin

To probe and optimize the obtained homology models of PCFT based on various X-ray-crystallography-derived templates by utilizing the substituted-cysteine accessibility method (SCAM), we used the functional Cys-less PCFT construct described above as template for generating 40 single-Cys mutants of PCFT (Figure 1). All constructs were confirmed by sequencing the entire coding region.

**Figure 3:**
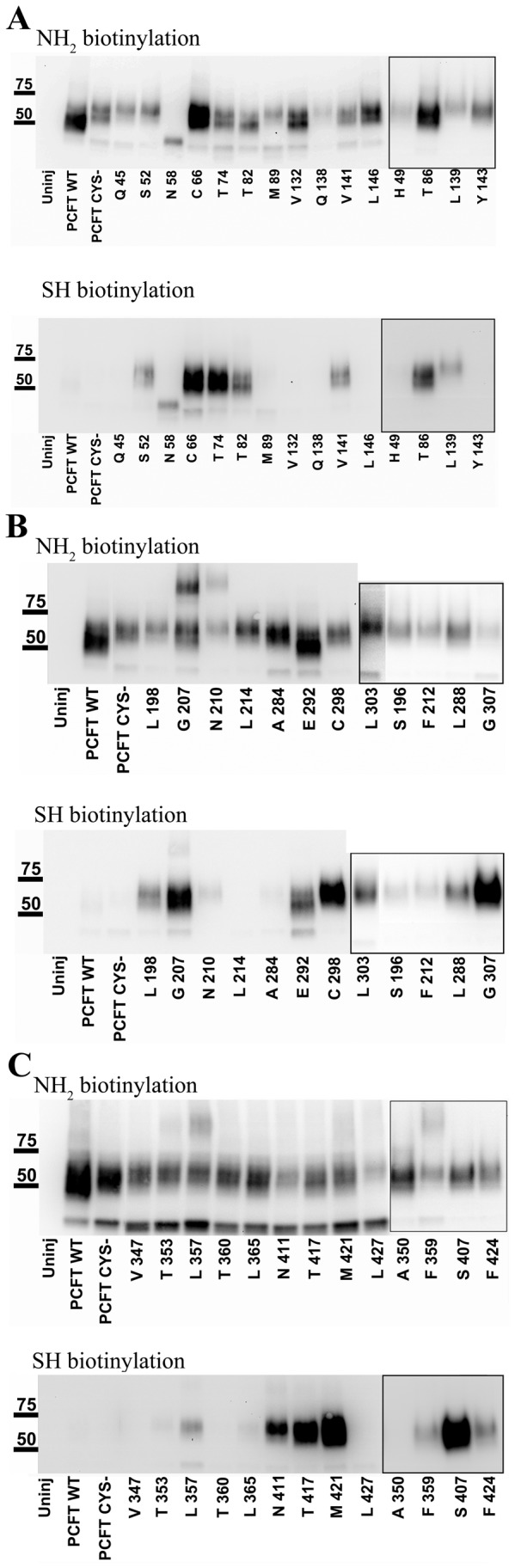
Accessibility of primary amines and Cys in HsPCFT wild-type, Cys-, and single-Cys constructs. (**A**) Fifteen residues were selected within and close to ECL1 and ECL2. (**B**) Six residues were investigated in each ECL3 and ECL4. (**C**) Seven residues each were probed in ECL5 and ECL6. NH2-biotinylation shows Western blot after plasma membrane fraction isolation following surface-NH_2_ biotinylation with sulfo-NHS-LC-biotin / avidin beads. Uninjected oocytes (uninj.) served as negative control, and PCFT V5 and PCFT CYS- injected oocytes served as positive control. SH-biotinylation shows Western blot after avidin isolation of MTSEA-biotin labeled Cys. Single-Cys engineered at positions indicated on top of the lanes. Equal amounts of protein samples were electrophoretically separated on 4-15 % precast gels, transferred to PVDF membranes, and probed with V5-HRP antibody (1/5,000 dilution in 5 % milk). The experiment was repeated on three different batches of oocytes and a representative Western blot is shown for each condition. Lanes in boxes are from separate experiments as additional positions were probed later.

It has been observed in previous studies that the plasma-membrane expression levels of different single-Cys mutants can vary widely [44]. We therefore used the plasma-membrane expression level to normalize our SCAM results. To evaluate the external accessibility of the single-Cys mytants we injected the corresponding RNA into *X. laevis* oocytes, and applied two procedures to each batch of oocytes in parallel: 1) we assessed the plasma-membrane expression level of each single-Cys construct individually, and 2) we probed the external accessibility of the engineered Cys. To investigate the plasma-membrane expression in *X. laevis* oocytes we used surface-biotinylation with sulfo-NHS-LC-biotin. Intact oocytes were treated with sulfo-NHS-LC-biotin. Due to the low pK_a_ of the sulfo moiety this biotinylation compound is charged at our experimental labeling pH, and therefore it is water soluble and unable to cross biological membranes. The reactive NHS moiety selectively labels primary amine groups as found in Lys sidechains and the N-terminal primary amine group. If labeling is performed on intact cells, only Lys exposed to the surface can be modified. Since modification introduces a biotin, biotinylated surface proteins including labeled PCFT can be isolated after cell lysis with avidin beads. Avidin-bead-purified proteins were separated by SDS-PAGE and PCFT detected with an antibody against the V5-epitope tag engineered at its C-terminus. PCFT wild-type and Cys-less PCFT are efficiently surface-biotinylated by this reagent and detected in Western blots (Figure 3). 

To probe the Cys accessibility a similar procedure was used after labeling with MTSEA-biotin. Under our reaction conditions MTSEA-biotin does not label PCFT wild-type or Cys-less PCFT (Figure 3). Additionally, in our hands MTSEA-biotin does not have access to internal Cys. Labeling of Cys in intracellular loops with MTSEA-biotin was only observed when oocytes were treated concomitantly with a permeabilizing agent like digitonin (10 µM, data not shown). 

Subsequently, we engineered Cys in all six extracellular loop-helix boundary areas, such that at least one position approximately at the center of the loop, at least one position at the start of both the adjoining N- and C-terminal α-helical segments, and at least one position inside both the adjoining N- and C-terminal α-helical segments were mutated individually to Cys. Consequently, for each of the six ECL regions, we tested 4-8 individual positions, for a total of 40 engineered Cys.

The plasma-membrane expression levels, determined by sulfo-NHS-LC-biotin labeling of 38 single-Cys constructs, were sufficiently high to infer further the accessibility of the engineered Cys with MTSEA-biotin. The plasma-membrane expression for the single-Cys constructs varied substantially (Figure 3). 

**Figure 4:**
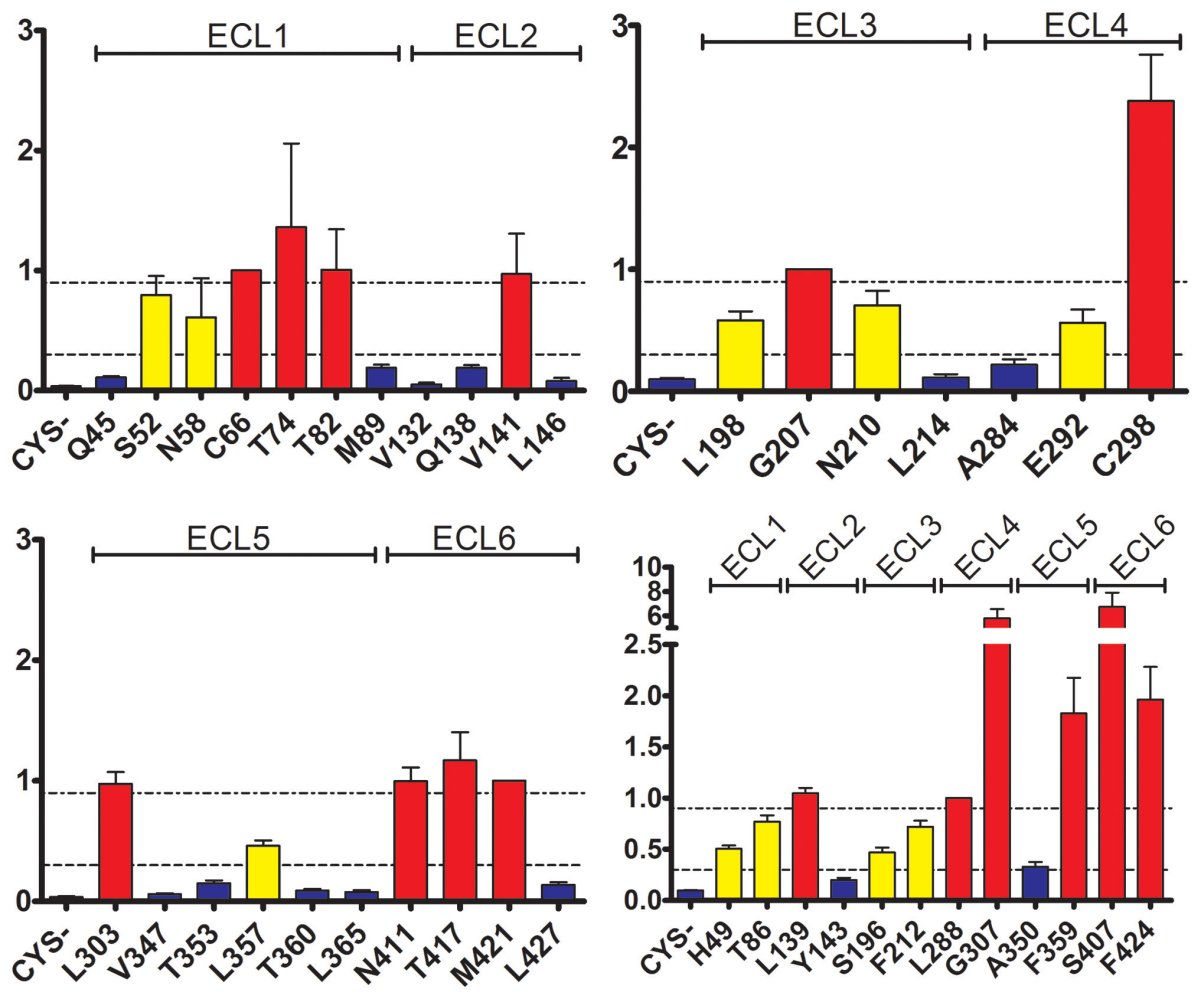
Color-coded Cys-accessibility normalized to NH_2_-accessibility. (A) ECL1 and ECL2. (B) ECL3 and ECL4. (C) ECL5 and ECL6. (D) Additional positions from all ECLs. Results shown in individual panels represent positions investigated on the same Western blot.

In the N-terminal domain of PCFT that comprises transmembrane segments I through VI, we tested a total of 21 positions. For the ECL1 region we probed nine positions that span a 45 residue segment, Q45, H49, S52, N58, C66, T74, T82, T86, and M89. For H49C the plasma-expression level was too low to make an assumption on the accessibility of the Cys. The other eight Cys constructs yielded good plasma-membrane expression (Figure 3). The ratio between Cys-labeling over Lys-labeling was quantified for all position and arbitrarily assigned to three bins, non-reactive, somewhat reactive, and highly reactive (Figure 4). Q45C and M89C were not accessible towards MTSEA-biotin. S52C, N58C, and T86C were somewhat accessible, and C66, T74C, and T82C were highly accessible (Figure 3, Figure 4). ECL2 region comprised six tested positions covering a 15 position amino acid stretch, V132, Q138, L139, V141, Y143, and L146. The plasma-membrane expression level of Q138C was too low to further investigate this position. Three positions, V132C, Y143C, and L146C were not accessible and two positions, L139C and V141C were highly accessible. Six positions as far apart as 18 positions were investigated in the ECL3 region, S196, L198, G207, N210, F212 and, L214. Here, one position, G207C, was highly accessible, four positions, S196C, L198C, N210C, and F212C, were somewhat accessible, and the L214C position was not accessible. 

Out of the C-terminal domain that contains transmembrane segments VII through XII, we assayed a total of 19 positions in the remaining three ECLs. In ECL4 we engineered six single-CYS spanning a 16 amino acid segment at the following positions: A284, L288, E292, C298, and G307. Only one position was not accessible, A284C, while four were highly accessible, L288C, C298C, and G307C, and E292C was somewhat accessible. In ECL5 seven positions along a 19 amino acid long stretch were tested, V347, A350, T353, L357, F359, T360, and L365. Five were not accessible, V347C, A350C, T353C, T360C, and L365C. L357C was somewhat assessable whereas F359 was highly accessible. In the last ECL, ECL6, a segment covering an 18-amino acid segment with six engineered Cys, S407, N411, T417, M421, F424, and L427, was investigated. All positions here, except L427C that was not accessible, were highly accessible. 

## Discussion

We utilized Cysteine scanning to investigate the loop-helix boundaries of the six extracellular loops in the 12-transmembrane segment MFS protein PCFT. In a previous study one residue from each extracellular loop was investigated to determine its external accessibility showing that C66, V141, G207, E292, L357 and T417 residues are externally accessible as these were labeled by MTSEA-biotin [45]. The experimental results in this study further substantiate the previously established extracellular loops of PCFT. The present study additionally extends the experimentally-derived accessibility of the extracellular surface of PCFT by firstly studying 40 instead of only 6 positions, and secondly quantifying the extent of accessibility by applying a ratiometric approach, i.e., Cys-labeled over Lys-labeled. The positions were chosen such that the substitution of the endogenous residues by Cys was a conserved mutation.

The reactivity of individual Cys may be influenced by a variety of factors to include: access to the bulk solution and the pKa of the respective Cys. Access to the bulk solution is influenced by steric factors as well as the electrostatic environment along the pathway to the Cys. It is expected to be very high at the protein surface and in large water-filled cavities. The pKa of a Cys in a protein environment is crucial, since MTS reagents react 10^9^ times faster with deprotonated thiolates over thiol Cys [46]. Most Cys SH have a pKa of 8.5 to 9, however, closely-apposing positively-charged residues and appropriately oriented aromatic residues can lower it by five orders of magnitude, to as low as 3.5 [47,48]. Additionally, lipid-buried Cys are not expected to ionize to a significant extend and are much less likely to react.

The aim of our study was to investigate the extent of accessibility of residues in the extracellular ends of transmembrane helices and extracellular loops towards the extracellular milieu using the membrane impermeable Cys-modifying agent MTSEA-biotin. All Cys were engineered in a Cys-less background construct that had all seven native Cys replaced by Ser and that - when injected into oocytes - exhibited WT PCFT-like transport of [^3^H]folic acid at pH 5.5 (Figure 2). Many membrane proteins have been prepared as Cys-less versions with similar to wildtype functionality for Cys-scanning methods [14,49-53]. However, instances where a Cys-less construct was non-functional or the Cys-substituting amino acids had to be optimized have also been reported. For PCFT it has been shown previously that Cys-less PCFT translocates folic acid substrates similarly to wildtype [15,16]. Only two out of the seven Cys of PCFT are involved in disulfide bond formation [15]. Since it has been shown that the plasma-membrane expression of Cys-less constructs can be decreased, we have normalized Cys-accessibility as determined with MTSEA-biotin in our present study against plasma-membrane expression levels [54]. 

**Figure 5:**
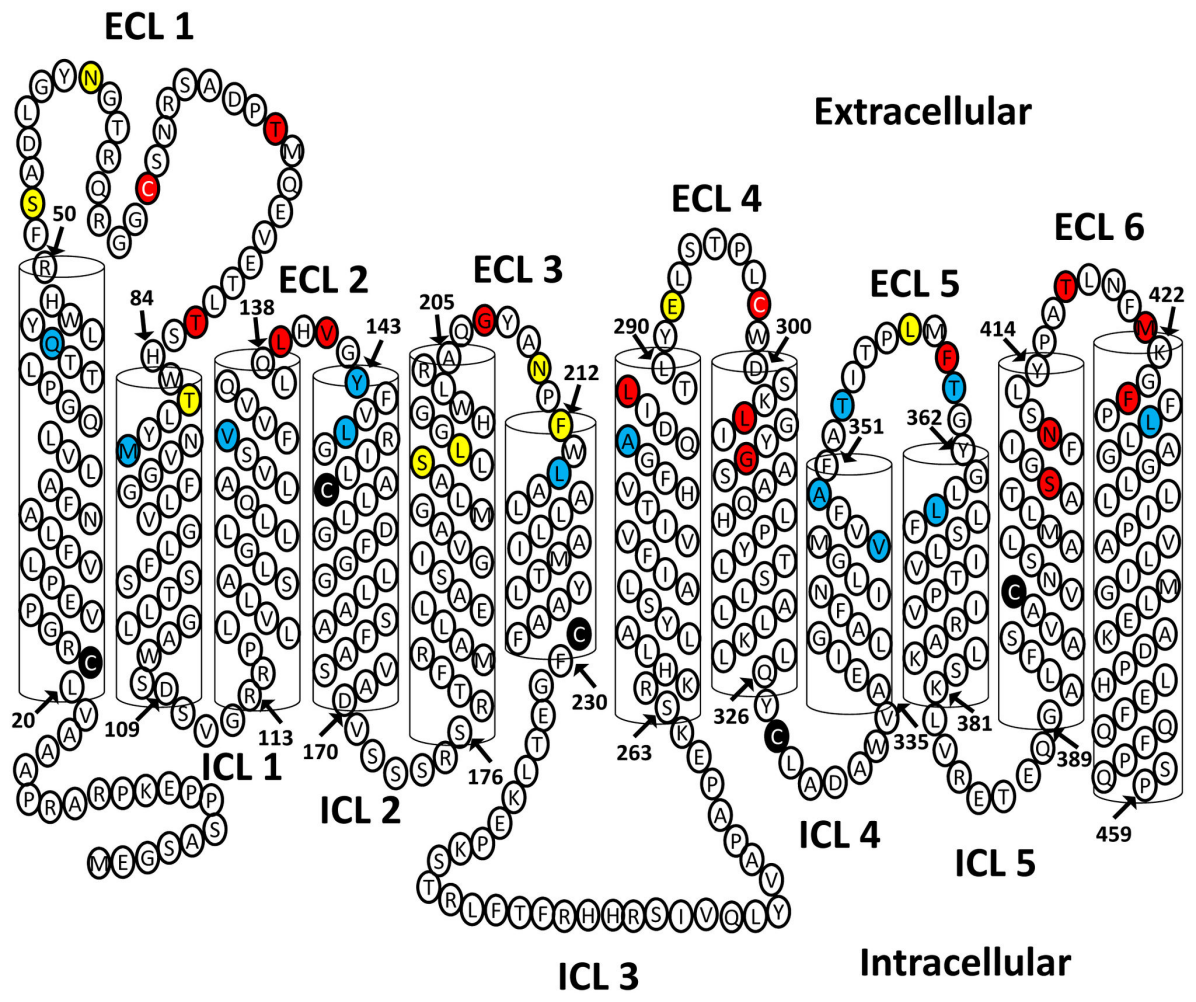
Color-coded Cys-accessibility normalized to NH_2_-accessibility plotted on two-dimensional model.

A large extracellular loop was predicted between TM1 and TM2 (Figure 5) [45,55,56]. Indeed, we found that 5 residues, from a stretch of a total of 34 amino acids, are Cys-accessible. On both the N- and C-terminal ends we found one position that was not accessible. Likely, these two positions are part of the respective α-helical segments of TM1 and TM2 where access to the bulk solution is limited either by compact packing with neighboring amino acids or lipids. The loop between TM3 and TM4 is very short, and two close-by positions that are highly accessible (L139C and V141C) are framed by inaccessible residues in close proximity. S196C is accessible from the extracellular environment even though it is positioned at 1/3 of the distance from the start of transmembrane segment 5. Similarly, L198, that is almost one α-helical turn towards the extracellular environment, is also accessible. S196C may be facing the proposed hydrophilic substrate pathway, and L198C may be at the lipid-protein interface in the head-group region. 

Importantly, we identified one unusual stretch of highly-accessible engineered Cys (S407-N411-T417-M421-F424). The first three are located on the extracellular end of TM11, and the latter two on the extracellular end of TM12. The location of S407C and N411C is below the bilayer level; however, these positions face the center of our PCFT model based on GlpT, and are likely lining the extracellular mouth of PCFT in the outward-facing state. A recent report investigated P425 extracellular accessibility with MTSEA-biotin and found that it is not accessible [57]. The neighboring residue (F424), is labeled by MTSEA-biotin, proving its accessibility to the extracellular environment. 

Overall, the extracellular water-accessibility of specific positions that are part of loop-helix regions on the extracellular surface of PCFT was determined. The loop between TM1 and TM2 is the largest extracellular loop, extending approximately from S42 to T82. Additionally, several residues that we predicted to be below the plasma membrane level based on our initial homology model were also labeled by MTSEA-biotin, likely because their side chains are accessible to potential hydrophilic substrate or proton-translocation pathway(s) at the center of PCFT. 

**Figure 6:**
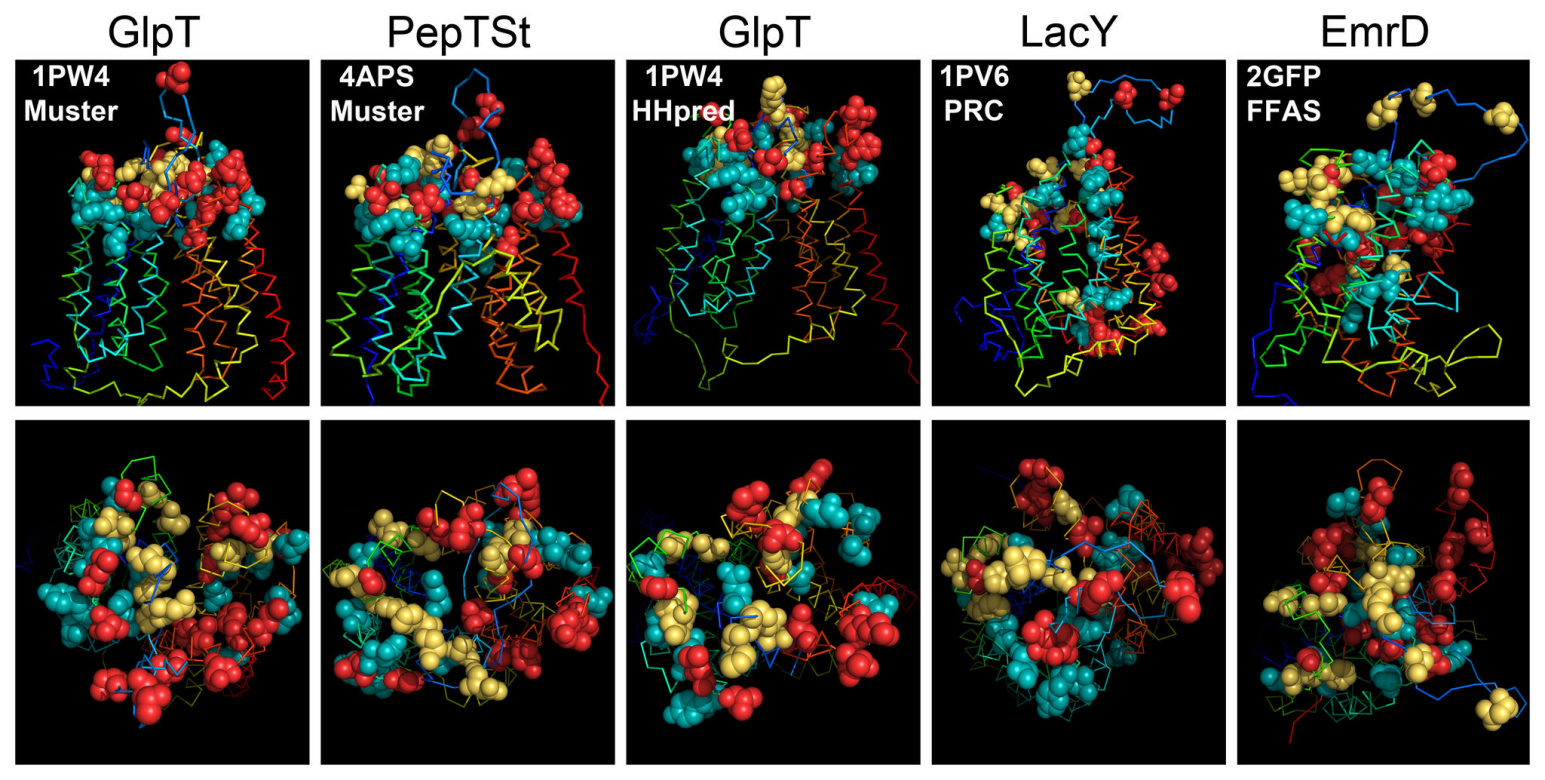
Three-dimensional models showing color-coded Cys-accessibility normalized to NH_2_-accessibility. Gene names are given above the respective panels, and pdb # and algorithm inside the top left corner of the upper panel for each model. View normal to the membrane in top row and from extracellular side in bottom row. The figures were prepared using the PyMOL Molecular Graphics System, Version 1.5.0.4 Schrödinger, LLC.

Our ratiometric approach allows to rationally investigate homology models generated by server-based structure-prediction methods that identify distant homologues within the PDB database. If we simply color-code the three ratio-bins and apply this color code to the investigated 40 residues in the 9 homology models we generated with HHpred/MODELLER and LOMETS/MODELLER, it is immediately obvious that several models contradict our experimental observation, since the colors seem to be randomly distributed over the entire transmembrane depth of the modeled PCFT (Figure 6). For some models, however, the experimentally determined highly-accessible residues are at the extracellular protein surface, either because they are at local extracellular pikes of the protein, or directly lining a large cavity found in the extracellular-facing center of the protein, whereas somewhat accessible positions are partially buried, and non-accessible positions are in fact buried. The models based on the templates GlpT and PepT_St_ best reflect our current results. 

Further experiments are needed for rational improvement of the alignments used for generating the models, for example with MODalign. Even though the sequence identity between PCFT and the template transporters with presently known X-ray structures is 14% or less at present, the core region is expected to be structurally well conserved with an RMSD for backbone atoms within almost atomic resolution, making the predicted structure of this core region more reliable. By experimentally probing the more flexible ECL regions and in the future also ICL regions, and further experimentation to delineate the exact vertical alignment between individual TMs it should be possible to generate a homology model with a precision within the current X-ray structure determination range for membrane proteins. A verified model will be of great use for future studies investigating residues involved in substrate and proton translocation as well as mechanistic studies.
